# Phenolic Profiling of Some Bryophyte Species: In Vitro and In Silico Assessment of Their Antimicrobial Potentials

**DOI:** 10.3390/molecules31152632

**Published:** 2026-07-28

**Authors:** Zafer Çambay, Harun Uslu, Ebru Coteli, Bünyamin Göktaş, Kevser Özdemir Bayçinar, Mustafa Yunus Emre, Muhammed Güngören, Mevlüt Alataş

**Affiliations:** 1 Department of Medical Services and Technique, Vocational School of Health Services, Fırat University, Elazığ 23119, Türkiye; 2 Department of Pharmaceutical Chemistry, Faculty of Pharmacy, Fırat University, Elazığ 23119, Türkiye; huslu@firat.edu.tr (H.U.); bgoktas@firat.edu.tr (B.G.); 3 Vocational School of Health Services, Kırşehir Ahi Evran University, Kırşehir 40100, Türkiye; e.coteli@ahievran.edu.tr; 4 Department of Pharmaceutical Chemistry, Graduate School, Anadolu University, Eskisehir 26470, Türkiye; 5 Department of Pharmacognosy, Faculty of Pharmacy, Fırat University, Elazığ 23119, Türkiye; kevser.ozdemir@firat.edu.tr; 6 Department of Nursing, Faculty of Health Sciences, Mardin Artuklu University, Mardin 47100, Türkiye; myemre@artuklu.edu.tr; 7 Department of Medical Services and Technique, Vocational School of Health Services, Mardin Artuklu University, Mardin 47100, Türkiye; muhammedgungoren@artuklu.edu.tr; 8 Tunceli Vocational School, Munzur University, Tunceli 62000, Türkiye; mevlutalatas@munzur.edu.tr

**Keywords:** bryophyte, phytochemistry, antimicrobial activity, molecular docking, phenolic acids

## Abstract

This study explores the phytochemical composition and antimicrobial potential of *Palustriella commutata*, *Philonotis calcarea*, *Cinclidotus riparius*, *Rhynchostegium riparioides*, *Plagiomnium ellipticum*, and *Porella platyphylla*, with a focus on bryophyte species phenolic constituents and biological activities. HPLC analysis identified seven phenolic acids (4-hydroxybenzoic acid, 2,5-dihydroxybenzoic acid, 3,4-dihydroxybenzoic acid, caffeic acid, vanillic acid, syringic acid, and *p*-coumaric acid) across all species in varying concentrations. In in vitro antimicrobial tests, the bryophyte extracts used showed significant activity against *Pseudomonas aeruginosa* (ATCC 9027), while exhibiting relatively lower inhibition against *Staphylococcus aureus* (ATCC 25923). The study showed that all species extracts exhibited comparable activity to erythromycin against *P. aeruginosa*, while *Cinclidotus riparius* and *Plagiomnium ellipticum* extracts showed no activity against *S. aureus*, and the remaining species exhibited lower activity compared to erythromycin. Molecular docking studies demonstrated strong binding affinities of these phenolics to the active site of the target macromolecule (PDB ID: 3FRQ), suggesting meaningful interactions with residues such as ARG122 and ASN123, similar to erythromycin binding patterns. The findings indicate that bryophyte-derived phenolic compounds possess significant antimicrobial potential and may serve as promising candidates for the development of novel bioactive agents. This study provides a broad framework for the insufficiently researched antimicrobial potential of bryophytes by establishing direct links between species-specific phenolic fingerprints, experimentally observed antibacterial activities, and predicted receptor-level interactions. Further complementary biological studies are needed to confirm and expand upon these results.

## 1. Introduction

The medicinal use of plants has extended from the dawn of humanity to the present day. Medicinal plants used as antimicrobial and antifungal agents have gained even greater importance today [1]. With the development of resistance to synthetically developed active substances and increasing mutations, the discovery of new active compounds is of paramount importance [2]. Bryophytes are a plant group found worldwide but have received little research attention due to a lack of public interest. Prior to this, bryophytes, which were not used as food, received insufficient attention [3]. However, their higher bioindicative values compared to the surrounding plants have prompted further research. The use of bryophytes in medicine has been prevalent since ancient times, particularly in Asia and during World War I. The abundant presence of flavonoids, glycosides, fatty acids, and volatile fatty acids in bryophytes’ composition has led to the belief that they may exhibit biological activities similar to other active compounds in plants [4,5,6].

The activities of bryophytes include cytotoxic, cardiotonic, antifungal, antimicrobial, pesticide, and plant growth regulator properties. Studies on bryophyte phytochemistry have reported the presence of terpenoids, flavonoids, phenolic compounds, acetogenins, vitamins, lipids, nitrogenous aromatic compounds and alkaloid derivatives in these plants. Phenolic compounds found in bryophytes may play an important role in terms of antimicrobial effects on microbial organisms [7,8,9,10,11,12,13,14,15,16,17,18,19].

Phenolic acids are the largest group of phenolic compounds formed as secondary metabolites [20]. They play a role in carrying out and regulating processes such as plant development, growth and pigmentation. Like other phenolic compounds, phenolic acids possess numerous biological activities that benefit human health. Previous studies have shown that various compounds in this class can exhibit biological effects such as antioxidant, anti-inflammatory, antimicrobial, antidiabetic, or anticarcinogenic activities [21,22,23,24,25]. It has been reported that the effects of phenolic acids, responsible for the antimicrobial activity observed in this study, are due to their lipophilicity [26]. Diffusion through cell membranes in a lipophilic form leads to protein denaturation. It has been observed that pH also affects the activity of phenolic acids; low pH makes cell membranes more permeable, leading to antimicrobial effects [27,28].

While the chemical profiles of tracheophytes have been extensively cataloged for pharmacological purposes, bryophytes remain a largely untapped reservoir, with existing reports often confined to broad, non-specific screenings. The distinctive novelty of this study lies in its multi-layered comparative approach: it bridges a major knowledge gap by establishing the individual phenolic acid fingerprints of six structurally and ecologically diverse, under-investigated bryophyte species (*Palustriella commutata* (Hedw.) Ochyra, *Philonotis calcarea* (Bruch & Schimp.) Schimp., *Cinclidotus riparius* (Host ex Brid.) Arn., *Rhynchostegium riparioides* (Hedw.) Cardot, *Plagiomnium ellipticum* (Brid.) T.J. Kop., and *Porella platyphylla* (L.) Pfeiff.), while systematically correlating these quantitative chemical findings with both in vitro growth inhibition profiles and in silico macromolecular docking behaviors against key human pathogens. Consequently, this work does not merely present a preliminary extract screening, but attempts to decode the structural–functional relationships governing their binding kinetics at the bacterial receptor level.

## 2. Results and Discussion

### 2.1. Phenolic Content Analysis

According to the results of the applied HPLC analysis, seven phenolic components were determined in *Palustriella commutata*, *Philonotis calcarea*, *Cinclidotus riparius*, *Rhynchostegium riparioides*, *Plagiomnium ellipticum* and *Porella platyphylla* extracts. The seven compounds obtained consist of 4-hydroxybenzoic acid, 2,5-dihydroxybenzoic acid, 3,4-dihydroxybenzoic acid, caffeic acid, vanillic acid, syringic acid and *p*-coumaric acid. The amounts of compounds obtained for *Palustriella commutata* were found to be 0.071–17.789 ppm, for *Philonotis calcarea* 0.085–4.41 ppm, for *Cinclidotus riparius* 0.029–2.576 ppm, for *Rhynchostegium riparioides* 0.059–15.323 ppm, for *Plagiomnium ellipticum* 0.063–14.385 ppm and for *Porella platyphylla* 0.13–74.258 ppm (Table 1 and Table 2).

### 2.2. In Vitro Analysis

**Figure 1:**
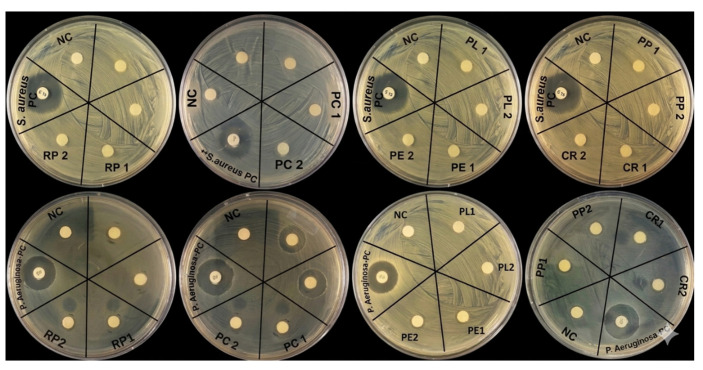
Petri dishes with zones of inhibition.

According to the results of the applied antimicrobial activity, *Cinclidotus riparius* and *Plagiomnium ellipticum* extracts showed no activity against *S. aureus* (ATCC 25923), while activity was observed against *Palustriella commutata*, *Philonotis calcarea*, *Rhynchostegium riparioides*, and *Porella platyphylla*, although this effect was lower compared to erythromycin. Against *P. aeruginosa* (ATCC 9027), activity was observed in all species extracts, and this effect was similar to that of erythromycin (Table 3). When the zone diameters observed on the aforementioned bacteria are evaluated both individually and against erythromycin, it can be said that the plant extracts are more effective against *P. aeruginosa* (ATCC 9027). When the zone diameters observed on the aforementioned bacteria are evaluated both individually and against erythromycin, it can be said that the plant extracts are more effective against *P. aeruginosa* (ATCC 9027) (Figure 1).

In Figure 1, *S. aureus*-PC represents the *Staphylococcus aureus* positive control, and *P. aeruginosa*-PC represents the *Pseudomonas aeruginosa* positive control. NC stands for negative control for all Petri dishes for both organisms. Here, the labels and slices on the Petri dish were marked using artificial intelligence (Nano Banana 2) and combined using a common background. The blank sections were excluded from the study and therefore were not labeled. The meanings of the other abbreviations used here can be explained as follows: PC: *Palustriella commutata*, PL: *Philonotis calcarea*, CR: *Cinclidotus riparius*, RP: *Rhynchostegium riparioides*, PE: *Plagiomnium ellipticum* and PP: *Porella platyphylla*.

### 2.3. Molecular Docking Studies

**Figure 2:**
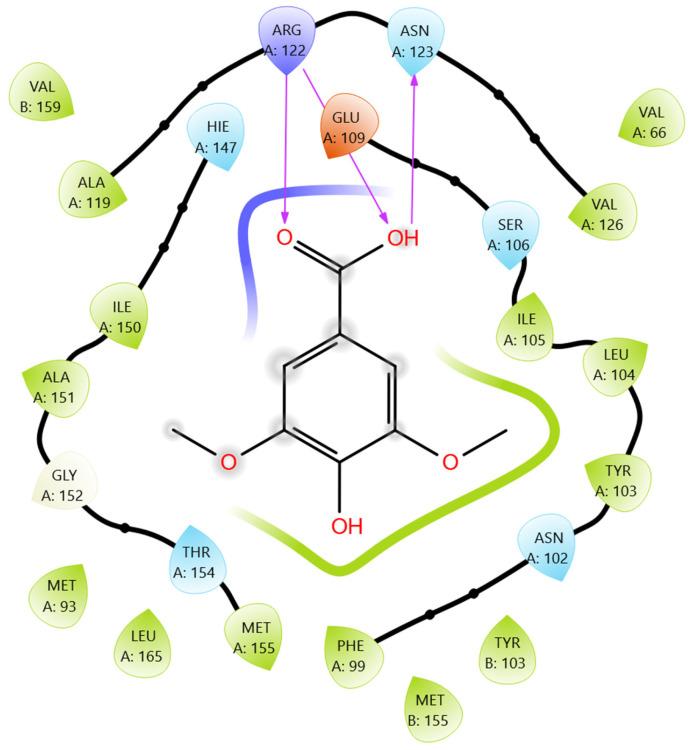
2D interaction diagram with 3FRQ for syringic acid.

**Figure 3:**
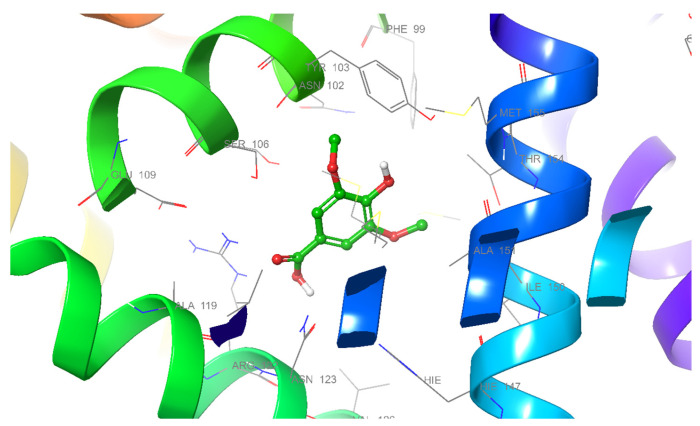
3D interaction diagram of 3FRQ with syringic acid.

**Figure 4:**
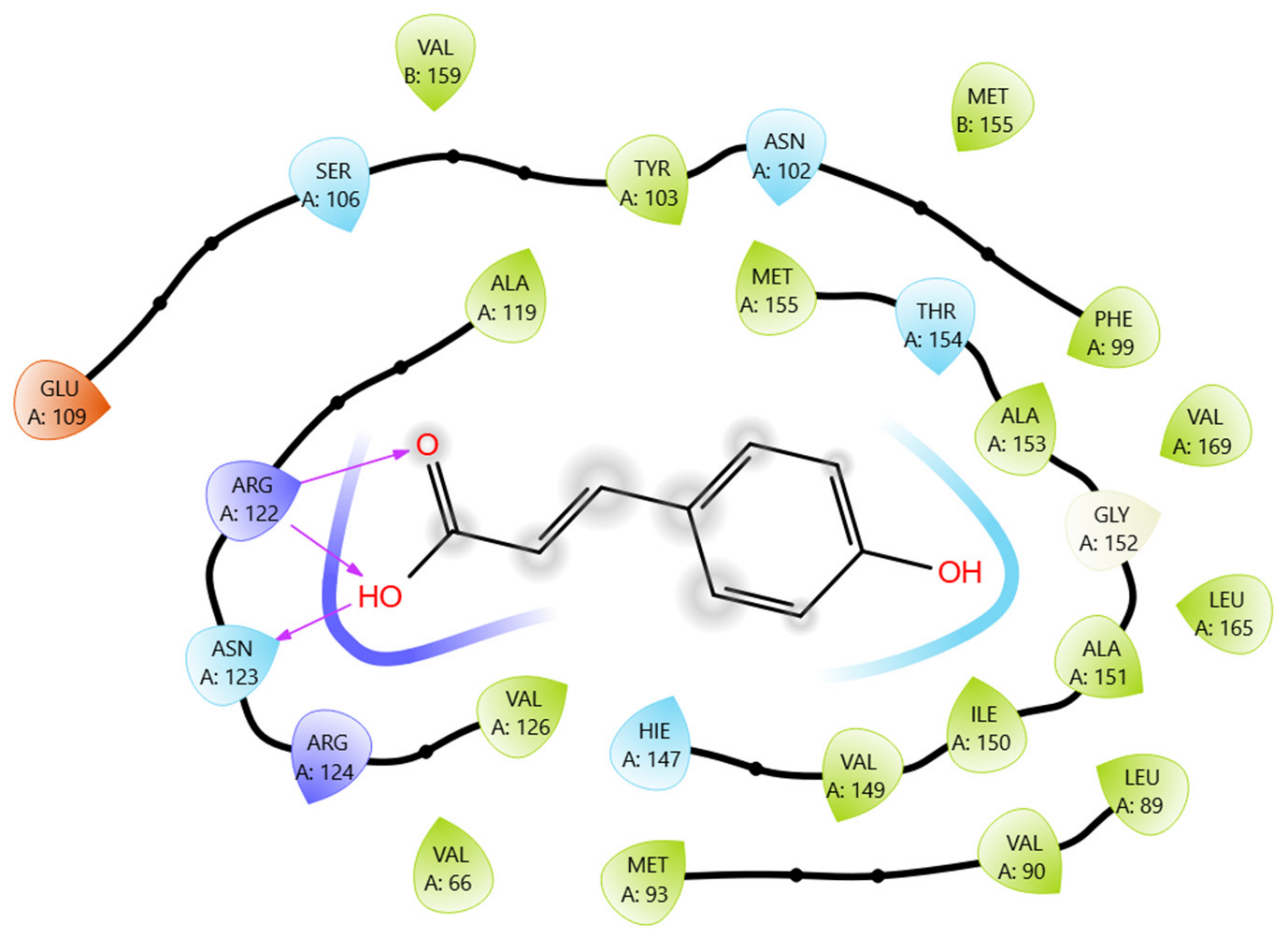
2D interaction diagram of 3FRQ with *p*-coumaric acid.

**Figure 5:**
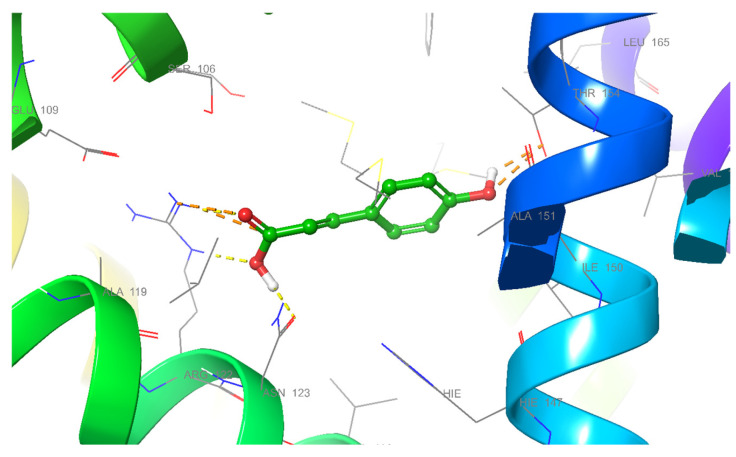
3D interaction diagram of 3FRQ with *p*-coumaric acid.

The active binding sites of the macromolecule (Pdb ID: 3FRQ) have been previously determined in the Protein Data Bank [41]. Docking studies were performed to see the interaction modes for all phenolic compounds obtained from plant extracts of *Palustriella commutata*, *Philonotis calcarea*, *Cinclidotus riparius*, *Rhynchostegium riparioides*, *Plagiomnium ellipticum* and *Porella platyphylla* with the active site of the macromolecule. Binding types and associated residues were generated in detail by Maestro Software 14.7 (Table 4, Figure 2, Figure 3, Figure 4 and Figure 5). The interaction modes with 3FRQ for 4-hydroxybenzoic acid, 2,5-dihydroxybenzoic acid, 3,4-dihydroxybenzoic acid, caffeic acid, vanillic acid, syringic acid, and *p*-coumaric acid were visualized in 2D and 3D with the Maestro Software [42].

Zheng et al.’s study revealed significant interactions between the macromolecule PDB ID: 3FRQ and the co-crystal ligand erythromycin. These included carbon-pi bonding with TYR69, hydrogen bonding with SER92, hydrogen bonding with ARG122, polar bonding with ASN123, hydrogen bonding with HIS147, polar bonding with ALA151, polar bonding with MET155, polar bonding with HOH199, and hydrogen bonding with HOH209, HOH232, HOH235, HOH287 and HOH300 [41].

### 2.4. Phytochemical Divergence and Mechanistic Antibacterial Interpretation

The quantitative distribution of the seven phenolic acids identified across the bryophytes studied (Table 2) reveals a chemotaxonomic differentiation. The most striking chemical difference observed is the exceptionally high concentration of syringic acid (74.258 ppm) found in *Porella platyphylla*, which is sharply contrasted with the minimal levels found in algae such as *Rhynchostegium riparioides* (0.059 ppm) and *Cinclidotus riparius* (0.955 ppm). Furthermore, the widespread presence of 2,5-dihydroxybenzoic acid and 3,4-dihydroxybenzoic acid in all species constitutes a key factor directly determining the biological properties of these crude extracts.

The in vitro bioassays exhibited a highly selective antimicrobial topography. While *Pseudomonas aeruginosa* (ATCC 9027) showed high susceptibility to all extracts, with inhibition zones of 9–10 mm closely approaching the clinical potency threshold of the reference antibiotic erythromycin (13 mm), *Staphylococcus aureus* (ATCC 25923) demonstrated complete resistance against *Cinclidotus riparius* and *Plagiomnium ellipticum* (N/A), and only minor susceptibility to the remaining species.

To decipher these outcomes at the sub-molecular level, in silico molecular docking targeted the macrolide biosensor protein MphR(A) (PDB ID: 3FRQ). The binding energies and structural orientations extracted via computational models strongly support the experimental data. *p*-Coumaric acid achieved the most thermodynamically stable binding configuration, exhibiting a docking score of −5.35 kcal/mol and a low estimated inhibition constant (Ki) of 119.21 µM, driven by a hydrogen-bonding network with ARG122 and ASN123 residues. Crucially, these specific amino acid coordinates (ARG122 and ASN123) represent the exact functional active site determined during the structural elucidation of crystalline erythromycin–macrolide biosensor complexes [41]. By translating macroscale inhibition zones into precise spatial amino acid interactions, these findings elevate the study from a descriptive chemical screening to a targeted, mechanistically backed evaluation of bryophyte-derived phenolics as viable scaffolds for natural antibiotic development.

## 3. Materials and Methods

### 3.1. Plant Material and Extraction Process

**Figure 6:**
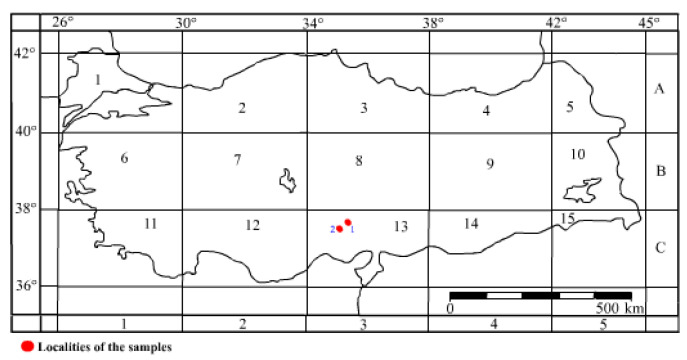
Henderson grid system of Türkiye and the localities where the bryophyte samples were taken.

The research materials were collected from Amcabey Alabalık Surrounding, Ulukışla (Niğde), located in C13 (Figure 6), and from Bolkar Mountains, Karagöl Surrounding Square, in Turkey, according to the Henderson [43] grid system. *Philonotis calcarea* is a taxon that likes open, moist and basic environments and spreads in calcareous areas of streams, lake edges and water sources, in wet soil, sand dune gaps, rocks and outcrops. *Palustriella commutata* is a taxon that loves basic and wet environments and is generally found between 0 and 900 m, in and next to calcareous springs and streams, on wet bedrock, in sand dunes, and on soil in open areas near water. *Cinclidotus riparius* is a taxon that spreads especially on the surface of rocks and in water in flood zones of rivers and likes wet, open and semi-neutral environments [44,45] (Table 5). The bryophyte specimens *Philonotis calcarea*, *Palustriella commutata* and *Cinclidotus riparius* used in the study are preserved in the bryophyte collection of Alataş (Tunceli) with the Herbarium numbers ALT 4640, ALT 4641, and ALT 4642, respectively. The yield of the extract was determined for *Rhynchostegium riparioides* (4.70%), *Plagiomnium ellipticum* (8.10%) and *Porella platyphylla* (9.90%).

**Figure 7:**
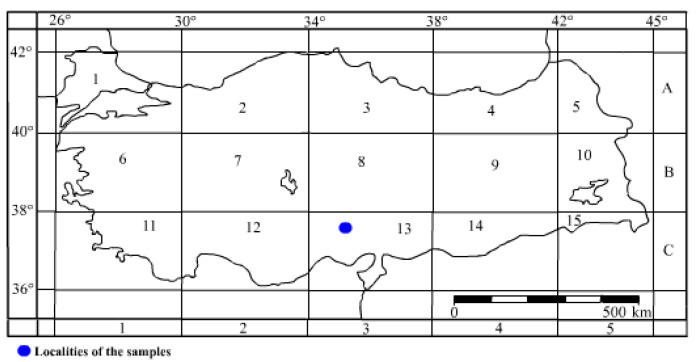
Henderson grid system of Türkiye and the locality from which the bryophyte samples were taken.

The research materials were collected from Bolkar Mountains, Kızılgöller surroundings, located in the C13 (Figure 7) Square in Turkey, according to the Henderson [43] grid system. *Rhynchostegium riparioides* is a taxon that thrives on rocks and tree roots inside and outside of submerged or fast-flowing streams and rivers, waterfalls, dams, locks, siphons, cliffs and quarries, and loves semi-neutral, moist and shaded environments. *Plagiomnium ellipticum* is a taxon that thrives in damp meadows and grasses, wet forests, and slightly acidic soils, and loves moist, shaded, and acidic environments. *Porella platyphylla* is a taxon that spreads on tree trunks and rocks in forest areas and likes semi-neutral, moist and shade environments [44,45] (Table 6). The bryophyte specimens *Rhynchostegium riparioides*, *Plagiomnium ellipticum* and *Porella platyphylla* used in the study are preserved in the bryophyte collection of Alataş (Tunceli) with the Herbarium numbers ALT 4643, ALT 4644, and ALT 4645, respectively.

The powdered plant was macerated with 70% ethanol for 3 days by rotating on a rotavapor for 1 h. The extraction was performed using a solvent-to-sample ratio of 10:1 (*v*/*w*). After filtering, it was evaporated to dryness on a rotavapor. The yield of the extract was determined for *Philonotis calcarea* (9.17%), *Palustriella commutata* (4.61%) and *Cinclidotus riparius* (7.70%). Then, 20 microliters of methanol was added to the weighed extracts, and stock concentrations were recorded as follows: *Philonotis calcarea*, 458.25 mg/mL; *Palustriella commutata*, 230.25 mg/mL; *Rhynchostegium riparioides*, 235 mg/mL; *Cinclidotus riparius*, 385.20 mg/mL; *Plagiomnium ellipticum*, 404.95 mg/mL; and *Porella platyphylla*, 495 mg/mL. These extracts were then bottled in airtight containers and stored at +4 °C until experiments were conducted.

### 3.2. Phenolic Content Analysis

HPLC-grade methanol and acetonitrile were purchased from Merck (Darmstadt, Germany), and all other chemicals were also purchased from Merck. Extract stocks of the aerial parts of *Palustriella commutata*, *Philonotis calcarea*, *Cinclidotus riparius*, *Rhynchostegium riparioides*, *Plagiomnium ellipticum*, and *Porella platyphylla*, prepared as previously described, were diluted with methanol (80%) and passed again through a 0.45 µm PDVF filter for the detection of some individual phenolic components. Samples and reference phenolic standards (4-hydroxybenzoic, 2,5-dihydroxybenzoic, 3,4-dihydroxybenzoic, caffeic, vanillic, and *p*-coumaric acids) to be used for qualitative/quantitative determination were injected in 20 µL volumes into the C-18 column (5 µm, 4.6 × 250 mm, GL Sciences, Tokyo, Japan) of a Waters (Milford, MA, USA) Alliance E2695 HPLC instrument. Phosphoric acid (solvent A; pH 2) and methanol:acetonitrile (solvent B; 90:10 V) were used as the mobile phase. The flow rate was set to 0.8 mL/min. The device was operated at 30 °C, and elution gradients were created with A–B solvent percentages for 2 min at 95–5%, 8 min at 75–25%, 10 min at 60–40%, 16 min at 50–50%, and 14 min at 0–100%. The system was then held steady for 10 min and returned to initial conditions after 13 min. The wavelength was set to 280 nm, and a DAD (diode array detector—Waters 2996, Milford, MA, USA) was used. The injection volume was 20 µL, and the temperature was 30 °C. Results were evaluated using the Empower 3 program. Calibration curves of reference standard substances were plotted, and qualitative/quantitative results were reported using chromatograms (Supplementary File) of the samples [46,47,48].

### 3.3. In Vitro Analysis

#### 3.3.1. Microorganisms Used in the Antimicrobial Activity Test

Gram-negative (*Pseudomonas aeruginosa* ATCC 9027) and Gram-positive (*Staphylococcus aureus* ATCC25923) bacteria were used to determine antimicrobial activity.

#### 3.3.2. Media Used in the Antimicrobial Activity Test and Their Preparation

Nutrient Broth (Merck, Darmstadt, Germany) and Nutrient Agar (Merck, Darmstadt, Germany) were used as liquid and solid media to cultivate the test bacteria used to determine antibacterial activity.

#### 3.3.3. Preparation of Nutrient Broth

Dehydrated medium was prepared using distilled water at 8.0 g/L, distributed into tubes, and sterilized in an autoclave at 121 °C for 15 min.

#### 3.3.4. Preparation of Nutrient Agar

The dehydrated medium was heated, dissolved in distilled water to a concentration of 20.0 g/L and sterilized in an autoclave at 121 °C for 15 min. After cooling to 45–50 °C, it was poured into sterile Petri dishes.

#### 3.3.5. Determination of Antimicrobial Activity

The disk diffusion method was used to determine antimicrobial activity according to the NCCLS guidelines. Bacteria taken from fresh cultures were incubated in Nutrient Broth at 37 °C until a turbidity equal to 0.5 McFarland was formed. Turbidity was measured spectrophotometrically and adjusted to between 0.08 and 0.10 at a wavelength of 625 nm. (The absorbance of a 0.5 McFarland standard should be between 0.08 and 0.10 at a wavelength of 625 nm in a spectrophotometer.) Subsequently, 0.1 mL of bacterial culture with appropriate turbidity was inoculated onto Nutrient Agar. Then, 6 mm sterile blank paper disks (Bioanalyse, Ankara, Turkey) were placed on the inoculated Petri dishes. Next, 10 μL of the prepared extracts (prepared in DMSO at an appropriate concentration) was absorbed onto the paper disks. Antimicrobial activity was determined by measuring the inhibition zone diameters formed after incubating the bacterial Petri dishes at 37 °C for 24 h. The same procedure was performed for the positive and negative controls (solvent). Each test was performed in triplicate, and the results are presented as the means (mm) of the inhibition zones ± the standard deviation [49].

#### 3.3.6. Positive Controls Used in Antimicrobial Activity Tests

Standard antibiotic disks were used as positive controls to examine the effects of these disks on the test microorganisms. The antibiotic disks used were erythromycin 15 μg/disk (Bioanalyse, Ankara, Türkiye).

### 3.4. Molecular Docking Studies

The phenolic contents of some bryophyte species were investigated by HPLC; the structures of these compounds were drawn by taking their smiles from PubChem (https://pubchem.ncbi.nlm.nih.gov). Energy minimization and molecular docking studies were conducted for all of these identified compounds. We used our previously reported procedure for molecular docking studies to assess 3FRQ antimicrobial activity. The interaction of the macrolide biosensor protein with erythromycin has been previously elucidated [41]. The macromolecule crystal structures were retrieved from the Protein Data Bank server (https://www.rcsb.org/, accessed on 7 November 2025) and optimized with Schrödinger Maestro 14.7 [42]. Molecular docking was performed with AutoDock Version 1.5.7 [50]. Erythromycin (Pdb ID: ERY), the macromolecule for 3FRQ, was re-docked into the target site of the macromolecule to validate the docking program and the RMSD value was found to be less than 1 (~0.7).

## 4. Conclusions

This study goes beyond determining antimicrobial activity, establishing a direct link between bryophyte phytochemistry and potential molecular mechanisms of action. By integrating HPLC-based phenolic profiling, in vitro antibacterial assays, and binding simulations within a single experimental framework, this research provides a comprehensive platform for the systematic evaluation of algae as a novel source of antimicrobial agents. In this study, a systematic comparative mapping of the individual phenolic acid profiles of six ecologically diverse bryophyte species (*Palustriella commutata*, *Philonotis calcarea*, *Cinclidotus riparius*, *Rhynchostegium riparioides*, *Plagiomnium ellipticum*, and *Porella platyphylla*) was successfully established alongside their in vitro and in silico antimicrobial characterizations. HPLC analysis revealed the presence of seven phenolic compounds (4-hydroxybenzoic acid, 2,5-dihydroxybenzoic acid, 3,4-dihydroxybenzoic acid, caffeic acid, vanillic acid, syringic acid, and *p*-coumaric acid) in varying concentrations among the bryophyte species studied, with *Porella platyphylla* exhibiting the highest phenolic content range (0.13–74.258 ppm). In vitro antimicrobial tests showed that most of the extracts exhibited activity against *Staphylococcus aureus* (ATCC 25923) and *Pseudomonas aeruginosa* (ATCC 9027), with generally stronger effects against *P. aeruginosa*. The quantitative HPLC analysis revealed a remarkable chemotaxonomic divergence, highlighted by an accumulation of syringic acid within the *Porella platyphylla*, suggesting highly specialized, species-specific metabolic adaptations. Molecular docking analyses revealed favorable interaction profiles between the identified phenolic compounds and the active site residues of the target protein (ARG122 and ASN123). Docking studies provided detailed information on binding modes and interacting residues, supporting the potential antimicrobial contribution of the identified phenolic acids. Overall, this study contributes to the growing body of knowledge about bryophyte phytochemistry and highlights the potential of these understudied plants as a source of biologically active compounds. Expanding analytical phytochemical profiling and conducting further biological studies may reveal additional therapeutic potentials of bryophyte-derived phenolics and support future applications in antimicrobial drug discovery.

## Figures and Tables

**Table 1:** Chemical structures of some phenolic acids investigated in plant extracts of *Palustriella commutata*, *Philonotis calcarea*, *Cinclidotus riparius*, *Rhynchostegium riparioides*, *Plagiomnium ellipticum*, and *Porella platyphylla*.

Comp.	Chemical Structure of Compounds
**1**	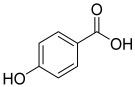 4-Hydroxybenzoic acid
**2**	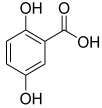 2,5-Dihydroxybenzoic acid
**3**	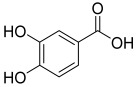 3,4-Dihydroxybenzoic acid
**4**	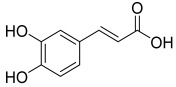 Caffeic acid
**5**	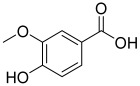 Vanillic acid
**6**	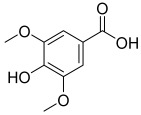 Syringic acid
**7**	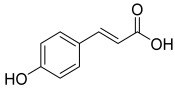 *p*-Coumaric acid

**Table 2:** Some chemical compositions of phenolic compounds obtained from plant extracts of *Palustriella commutata*, *Philonotis calcarea*, *Cinclidotus riparius*, *Rhynchostegium riparioides*, *Plagiomnium ellipticum*, and *Porella platyphylla* in ppm.

Comp.	*Palustriella* *commutata*	*Rhynchostegium* *riparioides*	*Porella* *platyphylla*	*Plagiomnium* *ellipticum*	*Philonotis* *calcarea*	*Cinclidotus* *riparius*
**1**	1.517	15.323	1.911	0.38	4.41	0.346
**2**	0.328	1.775	2.984	2.204	2.811	2.576
**3**	17.789	1.654	1.3	3.009	1.623	0.822
**4**	1.017	0.25	0.204	0.071	0.336	0.029
**5**	0.452	0.107	0.876	2.51	1.529	0.056
**6**	0.503	0.059	74.258	14.385	1.909	0.955
**7**	0.071	0.106	0.13	0.063	0.085	0.054

**1**: 3,4-dihydroxybenzoic acid; **2**: 4-hydroxybenzoic acid; **3**: 2,5-dihydroxybenzoic acid; **4**: caffeic acid; **5**: vanillic acid; **6**: syringic acid; **7**: *p*-coumaric acid. The phenolic compounds identified in this study have important biological functions. 4-Hydroxybenzoic acid has been reported to have antimutagenic, antibacterial, antialgal, and antifungal activity [29]. 2,5-Dihydroxybenzoic acid is one of the plant antioxidants that are essential in the medical, pharmaceutical, and food industries [30,31,32]. Studies have reported that 3,4-dihydroxybenzoic acid has anti-inflammatory, antioxidant, and neuroprotective properties [33,34]. Caffeic acid has antioxidant, antiviral, anticancer, and anti-inflammatory activities that reduce oxidative stress [35]. Vanillic acid is a nutraceutical molecule due to its antimicrobial, antioxidant, anti-inflammatory [36], anti-tumor, and anti-mutagenic properties [37]. Syringic acid exhibits beneficial properties such as being antimicrobial, antioxidant, anti-inflammatory, antidiabetic, and anticancer [38]. *p*-Coumaric acid has strong antioxidant [39], antimicrobial, and other biological properties [40]. The discovery of natural medicinal substances derived from plants is of enormous importance. It can be said that the bryophytes in this study contain pharmacologically important molecules.

**Table 3:** Inhibition zone diameters created by extracts (mm).

Plant Extracts	*S. aureus* (ATCC 25923)	*P. aeruginosa* (ATCC 9027)
*Palustriella commutata*	9 ± 0.5	10 ± 0.0
*Porella platyphylla*	8 ± 0.0	10 ± 0.8
*Cinclidotus riparius*	N/A	9 ± 0.0
*Philonotis calcarea*	9 ± 0.0	10 ± 0.5
*Plagiomnium ellipticum*	N/A	10 ± 0.5
*Rhynchostegium riparioides*	9 ± 0.0	10 ± 0.0
Erythromycin (15 µg/disk)	22 ± 0.4	13 ± 1.07

N/A: Not/activity.

**Table 4:** Molecular docking scores, interaction types, and estimated inhibition constants of phenolic compounds obtained from plant extracts of *Palustriella commutata*, *Philonotis calcarea*, *Cinclidotus riparius*, *Rhynchostegium riparioides*, *Plagiomnium ellipticum*, and *Porella platyphylla*.

Comp.	AutoDock Results		
	H-Bond Interacting Residues	Estimated Inhibition Constant, Ki	Best Docking Score
**1**	-	283.21 µM	−4.84
**2**	ARG122 ASN123	239.95 µM	−4.94
**3**	ASN102 ARG122 ASN123 ALA151	421.85 µM	−4.60
**4**	ASN102 ASN123 HIE147	1.09 mM	−4.04
**5**	ASN102 ASN123	323.59 µM	−4.76
**6**	ARG122 ASN123	249.07 µM	−4.92
**7**	ASN103 ARG122 ASN123	119.21 µM	−5.35

µM: micromolar, mM: millimolar, docking score: estimated free energy of binding (kcal/mol), **1**: 3, 4-dihydroxybenzoic acid; **2**: 4-hydroxybenzoic acid; **3**: 2,5-dihydroxybenzoic acid; **4**: caffeic acid; **5**: vanillic acid; **6**: syringic acid; **7**: p-coumaric acid.

**Table 5:** Locality information for *Philonotis calcarea*, *Palustriella commutata*, and *Cinclidotus riparius*.

Locality	Altitude (m)	Date	GPS Record
Amcabey Alabalık Surroundings, Ulukışla, Niğde	1216	13 September 2024	34°41′28.80″ E, 37°28′47.27″ N
Bolkar Mountains, Karagöl Surrounding	2598	12 September 2024	34°33′25.77″ E, 37°24′13.16″ N

**Table 6:** Locality information for *Rhynchostegium riparioides*, *Plagiomnium ellipticum*, and *Porella platyphylla*.

Locality	Altitude (m)	Date	GPS Record
Kızılgöller Surroundings	2750	01 September 2023	34°21′38″ E, 37°15′41″ N
Bolkar Mountains, Karagöl Surrounding	2598	12 September 2024	34°33′25.77″ E, 37°24′13.16″ N

## Data Availability

Dataset available on request from the authors.

## Supplementary Materials

The following supporting information can be downloaded at https://www.mdpi.com/article/10.3390/molecules31152632/s1: Figure S1: Chromatograms of phenolic compounds detected by HPLC instrument.
