# Supplementary material for: Phenolic Profiling of Some Bryophyte Species: In Vitro and In Silico Assessment of Their Antimicrobial Potentials
Source: Molecules. 2026 Jul 28;31(15):2632. doi: 10.3390/molecules31152632 (PMC13467868; doi:10.3390/molecules31152632)

Figure S1: Chromatograms of phenolic compounds detected by HPLC instrument. (PC: *Palustriella commutata*, PL: *Philonotis calcarea*, CR: *Cinclidotus riparius*, RP: *Rhynchostegium riparioides*, PE: *Plagiomnium ellipticum* and PP: *Porella platyphylla*).

CR

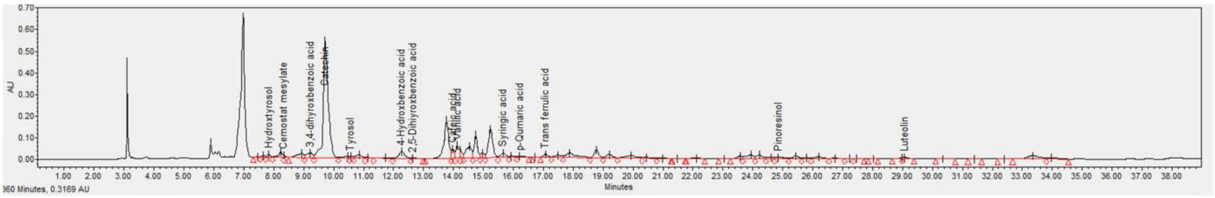

PL

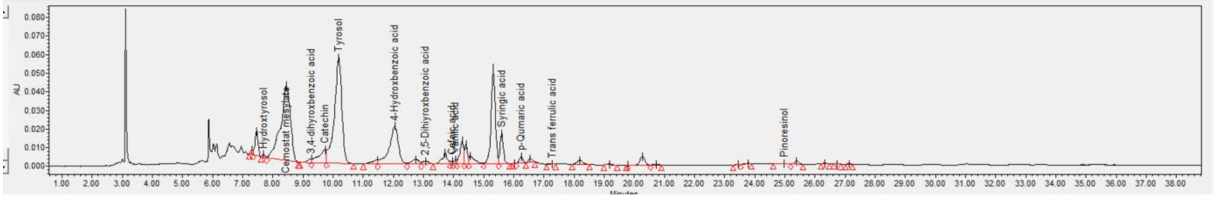

PE

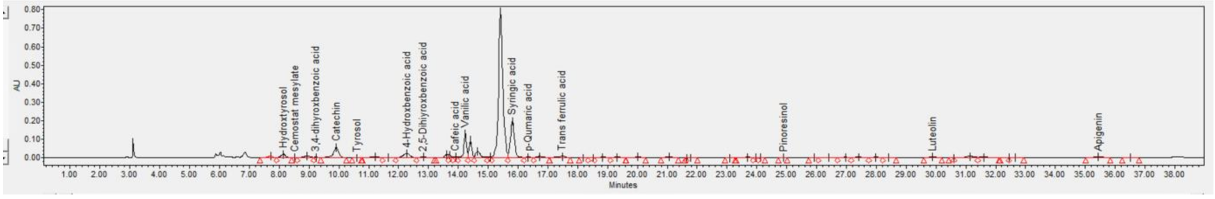

PP

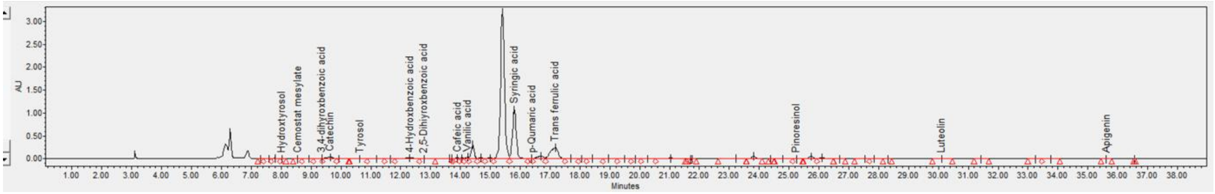

RP

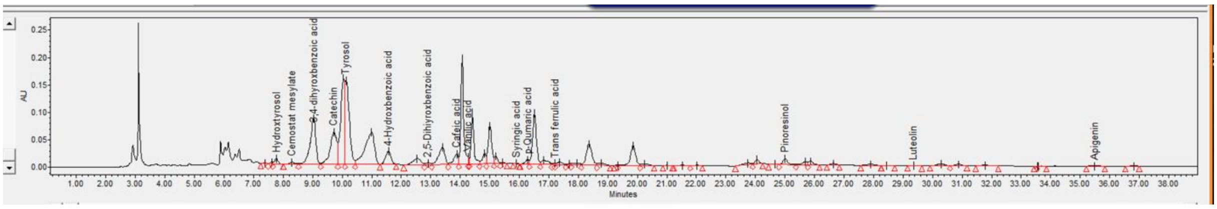

PC

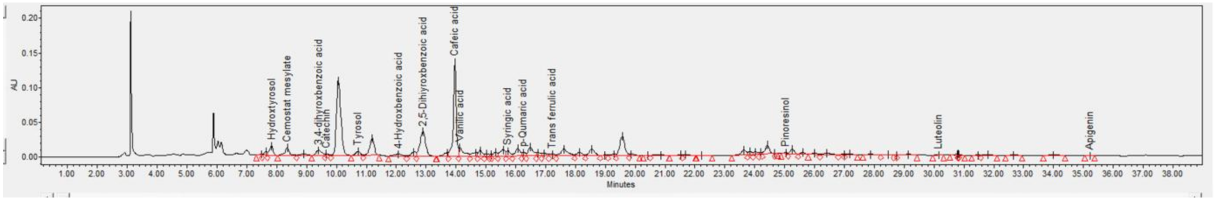

Supplement: Supplementary file 1 [file molecules-31-02632-s001.zip › molecules-4349721-supplementary.pdf]
